# 2-(4-Hy­droxy­phen­oxy)propanoic acid

**DOI:** 10.1107/S1600536810049469

**Published:** 2010-11-30

**Authors:** Zhun Gu, Hong-Sheng Jia, Wei Cheng

**Affiliations:** a Department of Biological and Chemical Engineering, Chien-shiung Institute of Technology, Taicang 215411, Suzhou, People’s Republic of China

## Abstract

In the title compound, C_9_H_10_O_4_, the carboxyl group is oriented at a dihedral angle of 84.6 (3)° with respect to the benzene ring. In the crystal, mol­ecules are linked *via* O—H⋯O hydrogen bonds.

## Related literature

For the synthesis and applications of the title compound, see: Qin *et al.* (2004 ▶).
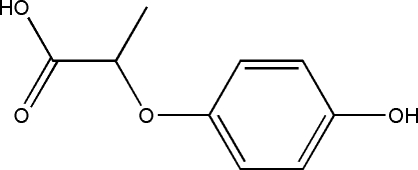


## Experimental

### 

#### Crystal data

C_9_H_10_O_4_
*M* *_r_* = 182.17
Monoclinic, 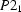

*a* = 6.205 (1) Å
*b* = 11.853 (2) Å
*c* = 6.716 (1) Å
β = 114.78 (3)°
*V* = 448.47 (15) Å^3^
*Z* = 2
Mo *K*α radiation
μ = 0.11 mm^−1^
*T* = 298 K
0.40 × 0.30 × 0.20 mm

#### Data collection

Enraf–Nonius CAD-4 diffractometer
924 measured reflections
924 independent reflections
829 reflections with *I* > 2σ(*I*)
*R*_int_ = 0.018
3 standard reflections every 200 reflections  intensity decay: none

#### Refinement

*R*[*F* ^2^ > 2σ(*F* ^2^)] = 0.040
*wR*(*F* ^2^) = 0.137
*S* = 1.02
924 reflections
121 parameters
3 restraints
H atoms treated by a mixture of independent and constrained refinement
Δρ_max_ = 0.24 e Å^−3^
Δρ_min_ = −0.28 e Å^−3^

### 

Data collection: *CAD-4 Software* (Enraf–Nonius, 1985 ▶); cell refinement: *CAD-4 Software*; data reduction: *XCAD4* (Harms & Wocadlo, 1995 ▶); program(s) used to solve structure: *SHELXTL* (Sheldrick, 2008 ▶); program(s) used to refine structure: *SHELXTL*; molecular graphics: *SHELXTL*; software used to prepare material for publication: *SHELXTL*.

## Supplementary Material

Crystal structure: contains datablocks I, global. DOI: 10.1107/S1600536810049469/xu5075sup1.cif

Structure factors: contains datablocks I. DOI: 10.1107/S1600536810049469/xu5075Isup2.hkl

Additional supplementary materials:  crystallographic information; 3D view; checkCIF report

## Figures and Tables

**Table 1:** Hydrogen-bond geometry (Å, °)

*D*—H⋯*A*	*D*—H	H⋯*A*	*D*⋯*A*	*D*—H⋯*A*
O1—H1*B*⋯O3^i^	0.85	1.94	2.733 (6)	154
O4—H4*B*⋯O1^ii^	0.85 (4)	1.84 (4)	2.679 (6)	166 (4)

Symmetry codes: (i) 
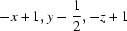
; (ii) 
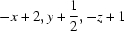
.

**Fig. 1.**
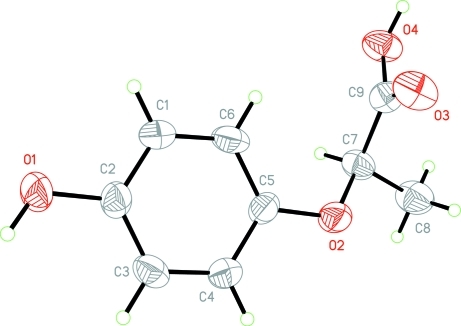
The molecular structure of the title molecule, with the atom-numbering scheme. Displacement ellipsoids are drawn at the 50% probability level.

**Fig. 2.**
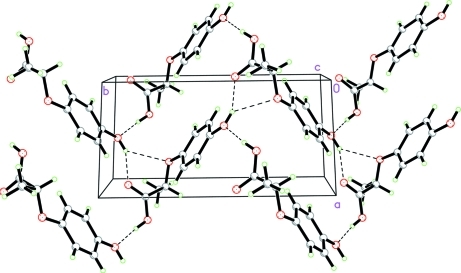
A packing diagram for (I). Hydrogen bonds are shown as dashed lines.

## supplementary crystallographic information

### Comment

The title compound, (I), is an important intermediate of the highly active herbicide *R*-clodinafop-propargyl (Qin *et al.*, 2004). We herein report its crystal structure.

The unit of the title compound, (I), (Fig. 1), contains one molecule and the bond lengths and angles (Table 1) are generally within normal ranges.

As can be seen from the packing diagram (Fig. 2), the intermolecular C—H···O hydrogen bonds (Table 2) link the molecules into three dimensional network, in which they may be effective in the stabilization of the crystal structure. Dipol-dipol and van der Waals interactions are also effective in the molecular packing.

### Experimental

The title compound was prepared by the literature method (Qin *et al.*, 2004). The crystals were obtained by dissolving the title compound (0.3 g) in ethanol (50 ml) and evaporating the solvent slowly at room temperature for 15 d.

### Refinement

The carboxyl H atom was located in a difference Fourier map and positional parameters were refined, U_iso_(H) = 1.5U_eq_(O). Other H atoms were positioned geometrically with C—H = 0.93-0.98 Å and O—H = 0.85 Å, and refined in ride mode with *U*_iso_(H) = 1.5U_eq_(C,O) for methyl H and hydroxyl H atoms and 1.2U_eq_(C) for the other H atoms.

### Crystal data

**Table:**

C_9_H_10_O_4_	*F*(000) = 192
*M**_r_* = 182.17	*D*_x_ = 1.349 Mg m^−^^3^
Monoclinic, *P*2_1_	Mo *K*α radiation, λ = 0.71073 Å
Hall symbol: P 2yb	Cell parameters from 25 reflections
*a* = 6.205 (1) Å	θ = 10–13°
*b* = 11.853 (2) Å	µ = 0.11 mm^−^^1^
*c* = 6.716 (1) Å	*T* = 298 K
β = 114.78 (3)°	Block, colorless
*V* = 448.47 (15) Å^3^	0.40 × 0.30 × 0.20 mm
*Z* = 2	

### Data collection

**Table:**

Enraf–Nonius CAD-4 diffractometer	*R*_int_ = 0.018
Radiation source: fine-focus sealed tube	θ_max_ = 25.9°, θ_min_ = 3.3°
graphite	*h* = −7→6
ω/2θ scans	*k* = 0→14
924 measured reflections	*l* = 0→8
924 independent reflections	3 standard reflections every 200 reflections
829 reflections with *I* > 2σ(*I*)	intensity decay: none

### Refinement

**Table:**

Refinement on *F*^2^	Primary atom site location: structure-invariant direct methods
Least-squares matrix: full	Secondary atom site location: difference Fourier map
*R*[*F*^2^ > 2σ(*F*^2^)] = 0.040	Hydrogen site location: inferred from neighbouring sites
*wR*(*F*^2^) = 0.137	H atoms treated by a mixture of independent and constrained refinement
*S* = 1.02	*w* = 1/[σ^2^(*F*_o_^2^) + (0.05*P*)^2^ + 0.6*P*] where *P* = (*F*_o_^2^ + 2*F*_c_^2^)/3
924 reflections	(Δ/σ)_max_ < 0.001
121 parameters	Δρ_max_ = 0.24 e Å^−^^3^
3 restraints	Δρ_min_ = −0.28 e Å^−^^3^

### Special details

**Table:**

Geometry. All e.s.d.'s (except the e.s.d. in the dihedral angle between two l.s. planes) are estimated using the full covariance matrix. The cell e.s.d.'s are taken into account individually in the estimation of e.s.d.'s in distances, angles and torsion angles; correlations between e.s.d.'s in cell parameters are only used when they are defined by crystal symmetry. An approximate (isotropic) treatment of cell e.s.d.'s is used for estimating e.s.d.'s involving l.s. planes.
Refinement. Refinement of *F*^2^ against ALL reflections. The weighted *R*-factor *wR* and goodness of fit *S* are based on *F*^2^, conventional *R*-factors *R* are based on *F*, with *F* set to zero for negative *F*^2^. The threshold expression of *F*^2^ > σ(*F*^2^) is used only for calculating *R*-factors(gt) *etc*. and is not relevant to the choice of reflections for refinement. *R*-factors based on *F*^2^ are statistically about twice as large as those based on *F*, and *R*- factors based on ALL data will be even larger.

### Fractional atomic coordinates and isotropic or equivalent isotropic displacement parameters (Å^2^)

**Table:**

	*x*	*y*	*z*	*U*_iso_*/*U*_eq_	
O1	0.4177 (6)	0.4704 (4)	0.2174 (6)	0.0527 (10)	
H1B	0.2918	0.4336	0.1940	0.079*	
O2	0.8378 (5)	0.7346 (3)	0.9648 (5)	0.0431 (9)	
O3	1.0305 (6)	0.9182 (3)	0.8307 (6)	0.0506 (10)	
O4	1.3803 (5)	0.8319 (3)	0.9703 (6)	0.0487 (9)	
C1	0.7253 (9)	0.5920 (5)	0.4402 (8)	0.0473 (13)	
H1A	0.7872	0.5876	0.3360	0.057*	
C2	0.5225 (8)	0.5344 (4)	0.4072 (8)	0.0381 (11)	
C3	0.4318 (8)	0.5400 (4)	0.5642 (9)	0.0401 (11)	
H3A	0.2959	0.4997	0.5449	0.048*	
C4	0.5441 (8)	0.6055 (4)	0.7487 (8)	0.0373 (10)	
H4A	0.4832	0.6092	0.8537	0.045*	
C5	0.7453 (8)	0.6655 (5)	0.7789 (7)	0.0375 (10)	
C6	0.8398 (9)	0.6568 (5)	0.6261 (8)	0.0505 (14)	
H6A	0.9797	0.6943	0.6483	0.061*	
C7	1.0878 (7)	0.7533 (4)	1.0603 (7)	0.0380 (11)	
H7A	1.1701	0.6824	1.0626	0.046*	
C8	1.1531 (10)	0.7924 (6)	1.2920 (8)	0.0538 (14)	
H8A	1.1081	0.7359	1.3698	0.081*	
H8B	1.0715	0.8616	1.2896	0.081*	
H8C	1.3214	0.8047	1.3643	0.081*	
C9	1.1595 (7)	0.8419 (4)	0.9378 (7)	0.0362 (10)	
H4B	1.448 (5)	0.883 (3)	0.928 (8)	0.054*	

### Atomic displacement parameters (Å^2^)

**Table:**

	*U*^11^	*U*^22^	*U*^33^	*U*^12^	*U*^13^	*U*^23^
O1	0.0343 (17)	0.065 (3)	0.059 (2)	−0.0158 (18)	0.0201 (16)	−0.030 (2)
O2	0.0322 (16)	0.055 (2)	0.0446 (18)	−0.0092 (16)	0.0189 (14)	−0.0137 (17)
O3	0.0359 (17)	0.050 (2)	0.061 (2)	0.0072 (17)	0.0158 (16)	0.0174 (19)
O4	0.0335 (17)	0.048 (2)	0.070 (2)	0.0068 (17)	0.0267 (16)	0.0125 (19)
C1	0.052 (3)	0.055 (3)	0.048 (3)	−0.015 (3)	0.034 (2)	−0.008 (3)
C2	0.030 (2)	0.036 (2)	0.046 (3)	0.001 (2)	0.015 (2)	−0.011 (2)
C3	0.029 (2)	0.035 (2)	0.056 (3)	−0.002 (2)	0.018 (2)	0.000 (2)
C4	0.035 (2)	0.041 (3)	0.040 (2)	0.001 (2)	0.0199 (19)	−0.001 (2)
C5	0.033 (2)	0.046 (3)	0.035 (2)	−0.002 (2)	0.0162 (18)	−0.011 (2)
C6	0.047 (3)	0.064 (4)	0.051 (3)	−0.025 (3)	0.031 (2)	−0.011 (3)
C7	0.031 (2)	0.043 (3)	0.037 (2)	−0.006 (2)	0.0114 (18)	0.001 (2)
C8	0.055 (3)	0.066 (3)	0.039 (3)	−0.014 (3)	0.019 (2)	0.000 (3)
C9	0.028 (2)	0.043 (3)	0.035 (2)	0.003 (2)	0.0115 (17)	0.001 (2)

### Geometric parameters (Å, °)

**Table:**

O1—C2	1.389 (6)	C3—H3A	0.9300
O1—H1B	0.8500	C4—C5	1.376 (7)
O2—C5	1.399 (5)	C4—H4A	0.9300
O2—C7	1.426 (5)	C5—C6	1.382 (6)
O3—C9	1.221 (6)	C6—H6A	0.9300
O4—C9	1.300 (5)	C7—C8	1.508 (7)
O4—H4B	0.85 (4)	C7—C9	1.511 (6)
C1—C2	1.366 (6)	C7—H7A	0.9800
C1—C6	1.382 (7)	C8—H8A	0.9600
C1—H1A	0.9300	C8—H8B	0.9600
C2—C3	1.389 (6)	C8—H8C	0.9600
C3—C4	1.378 (7)		
			
C2—O1—H1B	119.4	C1—C6—C5	119.7 (4)
C5—O2—C7	117.0 (4)	C1—C6—H6A	120.2
C9—O4—H4B	121 (3)	C5—C6—H6A	120.2
C2—C1—C6	120.9 (4)	O2—C7—C8	106.5 (4)
C2—C1—H1A	119.5	O2—C7—C9	112.1 (4)
C6—C1—H1A	119.5	C8—C7—C9	109.6 (4)
C1—C2—O1	117.8 (4)	O2—C7—H7A	109.6
C1—C2—C3	119.5 (4)	C8—C7—H7A	109.6
O1—C2—C3	122.7 (4)	C9—C7—H7A	109.6
C4—C3—C2	119.8 (4)	C7—C8—H8A	109.5
C4—C3—H3A	120.1	C7—C8—H8B	109.5
C2—C3—H3A	120.1	H8A—C8—H8B	109.5
C5—C4—C3	120.6 (4)	C7—C8—H8C	109.5
C5—C4—H4A	119.7	H8A—C8—H8C	109.5
C3—C4—H4A	119.7	H8B—C8—H8C	109.5
C4—C5—C6	119.5 (4)	O3—C9—O4	123.4 (4)
C4—C5—O2	116.2 (4)	O3—C9—C7	124.4 (4)
C6—C5—O2	124.3 (4)	O4—C9—C7	112.0 (4)
			
C6—C1—C2—O1	−179.6 (5)	C2—C1—C6—C5	−1.4 (9)
C6—C1—C2—C3	−0.8 (8)	C4—C5—C6—C1	2.9 (9)
C1—C2—C3—C4	1.5 (7)	O2—C5—C6—C1	−175.5 (5)
O1—C2—C3—C4	−179.8 (5)	C5—O2—C7—C8	−160.4 (5)
C2—C3—C4—C5	0.0 (7)	C5—O2—C7—C9	79.9 (6)
C3—C4—C5—C6	−2.2 (8)	O2—C7—C9—O3	26.7 (7)
C3—C4—C5—O2	176.3 (4)	C8—C7—C9—O3	−91.3 (6)
C7—O2—C5—C4	150.4 (4)	O2—C7—C9—O4	−157.2 (4)
C7—O2—C5—C6	−31.1 (7)	C8—C7—C9—O4	84.8 (5)

### Hydrogen-bond geometry (Å, °)

**Table:**

*D*—H···*A*	*D*—H	H···*A*	*D*···*A*	*D*—H···*A*
O1—H1B···O3^i^	0.85	1.94	2.733 (6)	154
O4—H4B···O1^ii^	0.85 (4)	1.84 (4)	2.679 (6)	166 (4)

Symmetry codes: (i) −*x*+1, *y*−1/2, −*z*+1; (ii) −*x*+2, *y*+1/2, −*z*+1.
